# Evaluation of the Effectiveness of Animated Images in First Aid for Infants with Foreign Body Airway Obstruction: A Simulation Study

**DOI:** 10.3390/jcm14082839

**Published:** 2025-04-20

**Authors:** Taekgeun Ohk, Junhwi Cho, Hyunseok Cho, Goeun Yang, Kicheol You, Taehun Lee

**Affiliations:** 1 Department of Emergency Medicine, College of Medicine, Kangwon National University, 24341 Chuncheon, Republic of Korea; otgotg11@gmail.com (T.O.); cjhemd@kangwon.ac.kr (J.C.); kickboi815@hanmail.net (H.C.); 2 Department of Emergency Medicine, Kangwon National University Hospital, 24289 Chuncheon, Republic of Korea; 3 Department of Pediatric Emergency, Kangwon National University Hospital, 24289 Chuncheon, Republic of Korea; 4 Department of Radiology, College of Medicine, Kangwon National University, 24341 Chuncheon, Republic of Korea; yangke00@hanmail.net; 5 Department of Radiology, Kangwon National University Hospital, 24289 Chuncheon, Republic of Korea; 6 Department of Emergency Medicine, Chuncheon Sacred Heart Hospital, Hallym College of Medicine, 24253 Chuncheon, Republic of Korea; emykc@hallym.or.kr

**Keywords:** airway obstruction, animated GIFs, emergency medical system, first aid, pediatric emergency medicine

## Abstract

**Background:** Foreign body airway obstruction is a sudden emergency that can occur unexpectedly in healthy people, leading to severe consequences if immediate first aid is not provided. Unlike the Heimlich maneuver for adults, the first aid for infant choking is less widely known and more complex, making it difficult to explain verbally. This study aimed to assess the efficiency of using an animated graphics interchange format (GIF) to teach first aid for infant choking due to foreign bodies. **Methods:** Eighty adults who had not received recent training in choking first aid within the last two years were randomly assigned to either the auditory (*n* = 40) or audiovisual (*n* = 40) groups. The participants were asked to perform first aid on an infant manikin under the guidance of a researcher using a smartphone in a separate room. The auditory group received verbal instructions only, while the audiovisual group received animated GIFs on their smartphones along with verbal instructions simultaneously. The entire process was recorded with two cameras, and two emergency physicians reviewed the videos to assess the adequacy of the first aid administered. **Results:** The “infant position”, “supporting arm posture”, and “head tilt” were more adequate in the audiovisual group. The Instruction Performance scores were higher in the audiovisual group. There was no significant difference in the time required to administer first aid between the two groups. **Conclusions:** Audiovisual guidance using animated GIFs has been shown to effectively enhance the adequacy of first-aid performance for infant airway obstruction caused by foreign bodies.

## 1. Introduction

Foreign body airway obstruction (FBAO) is a sudden emergency that can occur unexpectedly, especially during eating, even in healthy individuals. Delaying immediate emergency treatment for FBAO can lead to severe consequences, such as a vegetative state or death [1]. According to the most recent guidelines of the American Heart Association from 2020, in cases of severe airway obstruction in adults or children over 1 year of age, whether conscious or not, it is crucial to call “Emergency Medical Services (EMS)” immediately and perform abdominal thrusts repeatedly until the obstruction is cleared or the patient loses consciousness. For infants under 1 year old, alternating between five back blows and five chest thrusts is recommended [2].

Explaining the appropriate first aid for FBAO to an untrained individual over the phone can be challenging, and inappropriate first-aid measures may waste precious time in treating the patient [3,4]. While the Heimlich maneuver for adult FBAO is relatively well known, first aid for infant FBAO is less familiar to those without prior training.

In the United States, FBAO is a leading cause of accidental death among infants and the fourth leading cause of death among preschoolers aged 5 years or younger [5]. A study on airway obstruction time and prognosis in patients with FBAO reported that only 42% of bystanders provided accurate first aid for adult FBAO [1]. Igarashi et al. estimated that the rate of accurate first-aid provision for infants under 1 year old with FBAO may be even lower [3].

It is crucial to accurately replicate airway obstruction emergency treatment methods for effective first aid in cases of infant FBAO [6] and to explore how laypersons can better replicate complex infant FBAO first-aid methods. However, relevant studies in this field remain scarce.

Improving the accuracy and immediacy of first aid will help prevent serious complications, such as hypoxia and brain damage, in infants due to airway obstruction and improve overall survival.

To enhance both accuracy and immediacy, it is important to deliver first-aid information more quickly and precisely, which may be effectively achieved through visual-based interventions [7].

With advancements in communication technologies, numerous recent studies in the field of out-of-hospital cardiac arrest have explored the use of video recordings and real-time video calls to enhance the delivery of first aid. These studies have reported positive outcomes, demonstrating that the accurate implementation of cardiopulmonary resuscitation (CPR) in accordance with established guidelines contributes to improved survival rates. However, several limitations requiring further improvement have also been identified [8,9].

**Figure 1:**
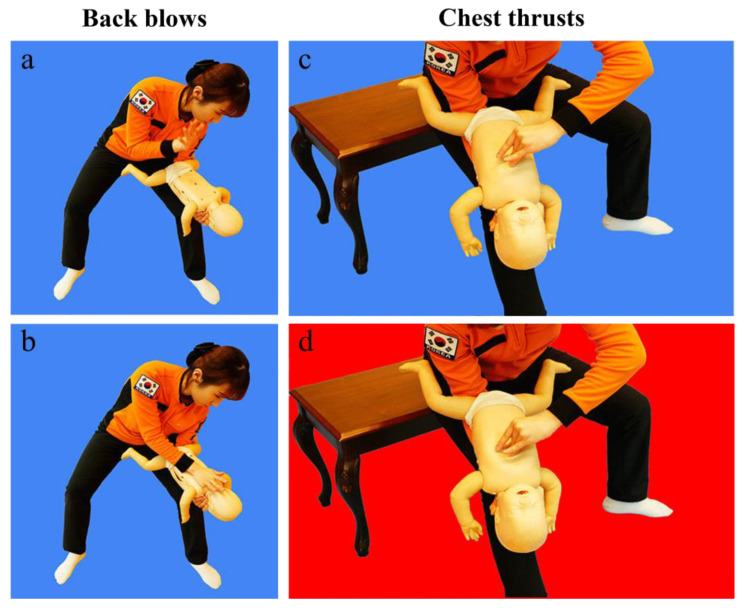
Small-packet animated graphics interchange formats (GIFs). The figure illustrates the sequential phases of the back blow (**a**,**b**) and chest thrust (**c**,**d**) maneuvers, respectively.

This study aimed to assess the impact of adding a visual aid (Figure 1) to standard auditory instructions provided by dispatchers over the phone for infant FBAO first aid on laypersons’ ability to replicate the standard infant FBAO first-aid position and follow instructions.

## 2. Materials and Methods

### 2.1. Study Design and Setting

This was a prospective, single-blind, randomized, and simulated study. The Consolidated Standards of Reporting Trials (CONSORT) reporting guideline was also followed [10]. The sample sizes were determined using G.power 3.1.9.7. The sample size was determined using a two-tailed test with an effect size (d) of 1.17, alpha of 0.05, and power of 0.95. This required 20 participants per group, totaling 40. The achieved power was 0.95. Eighty participants were randomly assigned to either the auditory group or the audiovisual group, with 40 participants in each group. The participants were blinded to the experiment, with only the instructions to “perform first aid as directed”. The participants were instructed to perform first aid for airway obstruction on an infant manikin (Baby Anne, Laerdal) in a given FBAO situation. Using their smartphones, the participants reported to a researcher acting as a dispatcher and followed the instructions provided by a research facilitator based on the 2020 American Heart Association Emergency Cardiovascular Care Guidelines. The research environment simulated an infant FBAO situation outside a hospital with activated EMS. The researcher guided the participants remotely using only a smartphone following the infant FBAO protocol from the Gangwon-do Fire Headquarters situation room (Appendix A).

### 2.2. Selection of Participants

**Figure 2:**
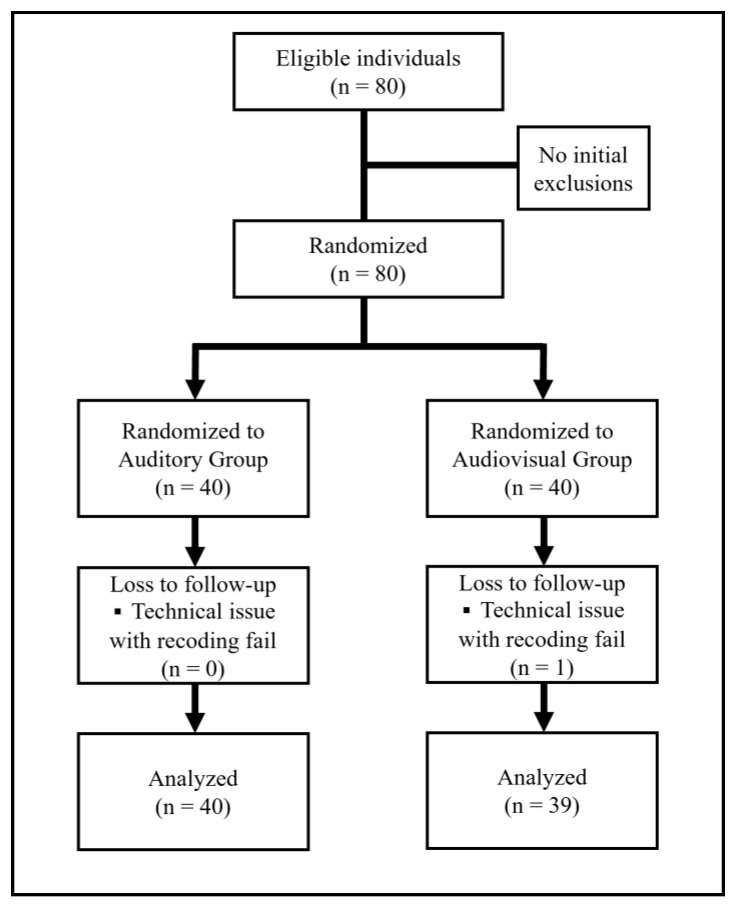
CONSORT flow diagram.

The study included 80 adult participants aged 19 to 65 who had not received cardiopulmonary resuscitation (CPR) and airway obstruction first-aid training in the past two years and voluntarily participated in this study. Children and elderly people inexperienced in using smartphones and those with disabilities that prevented them from performing infant FBAO first aid were excluded (Figure 2).

Participants were recruited through a public announcement. Before participation, the researcher explained the study in detail for approximately 30 min, and written informed consent was obtained.

### 2.3. Interventions

The two groups participated in the study under identical conditions, including the use of the same place and the same infant manikin. For the auditory group, the researcher delivered first-aid instructions solely through audio. In contrast, for the audiovisual group, the researcher sent a “visualized simple converted image” to the participants’ smartphones along with audio instructions on administering first aid. The “visualized simple converted image” was created in collaboration with the Gangwon-do Fire Department, Republic of Korea, in consultation with the authors. It consists of a series of two standardized infant FBAO first-aid images that create a dynamic, moving effect (Figure 1).

### 2.4. Randomization

The participants were unaware of the group they belonged until they entered the room, where they were randomly assigned to a group via a paper lottery. Paper slips representing each allocation group (auditory and audiovisual) were prepared in the same size and shape and thoroughly mixed to ensure equal opportunity. To ensure confidentiality, the lottery was conducted using a sealed, opaque container. Neither the researchers nor the participants knew the allocations until assignment. Randomization was performed by an independent research assistant. After the lottery, the participants opened the sealed envelope to check their assigned group.

### 2.5. Outcomes

The main study outcome was the scores for Instruction Performance (IP). The entire process was recorded using two video cameras, one positioned in front and one from the side. After the simulation experiment was completed, two emergency physicians reviewed the videos to assess the adequacy of first aid and IP. First-aid appropriateness was assessed based on 10 items, while IP was quantified as the “IP score” by summing up the scores of all items, with 1 point given for each appropriateness item. The time from when the dispatcher started instructing the participant on performing first aid to the participant executing the first back blow was defined as “time to the first back blow”. Additionally, the time from the start of instruction on performing first aid to the completion of five back blows and five chest thrusts was defined as “time to finish the first cycle” (Table 1).

### 2.6. Statistical Analysis

All statistical tests were two-tailed, and *p* <0.05 was considered statistically significant. All statistical analyses were conducted using the Statistical Package for the Social Sciences statistics version 25.0 (IBM Co., Armonk, NY, USA). Continuous variables were presented as the mean and standard deviation. Continuous and categorical variables were compared using the *t*-test and chi-squared test.

## 3. Results

### 3.1. General Characteristics

One participant in the audiovisual group was unable to be evaluated due to problems with the recorded video and was excluded from the study. Finally, the study included 40 and 39 participants in the auditory and audiovisual groups, respectively (Figure 2). In the auditory group, the mean age was 30.43 ± 3.11 years, there were 19 males, 15 participants had relevant training before, and the mean time since training was 4.88 ± 2.30 years. In the audiovisual group, the mean age was 30.46 ± 2.49 years, there were 13 males, 20 participants had relevant training before, and the mean time since training was 5.37 ± 2.41 years. There were no significant differences between the two groups in terms of sex, age, or prior training experience (Table 2).

### 3.2. Comparative Analysis of the Auditory and Audiovisual Groups

Regarding the adequacy of first aid, the “infant position”, “supporting arm posture”, “head tilt”, and “hand part for back blows” were more adequate in the audiovisual group than in the auditory group. The “number of chest thrusts” was more adequate in the auditory group than in the audiovisual group. The audiovisual group had higher IP scores than the auditory group [8.15 ± 1.31 vs. 6.13 ± 2.27, *p* < 0.001]. Regarding the time required for first aid, no significant differences were observed in all items between the two groups (Table 3).

### 3.3. Analysis According to Prior Relevant Training Experience

#### 3.3.1. Comparative Analysis of the Auditory and Audiovisual Groups with Prior Relevant Training

Regarding the adequacy of first aid, the “head tilt” and “number of back blows” were more adequate in the audiovisual group than in the auditory group. However, IP scores did not differ significantly between the auditory and audiovisual groups. Regarding the time required for first aid, no significant differences were observed in all items between the two groups (Table 4).

#### 3.3.2. Comparative Analysis of the Auditory and Audiovisual Groups Without Prior Relevant Training

Regarding the adequacy of first aid, the “infant position”, “supporting arm posture”, and “head tilt” were more adequate in the audiovisual group than in the auditory group. The “number of chest thrusts” was more adequate in the auditory group than in the audiovisual group. The audiovisual group had higher IP scores than the auditory group [8.05 ± 1.31 vs. 5.63 ± 2.24, *p* = 0.014]. Regarding the time required for first aid, no significant differences were observed in all items between the two groups (Table 4).

### 3.4. Analysis According to Sex

#### 3.4.1. Comparative Analysis of the Auditory and Audiovisual Groups Among Male Participants

Regarding the adequacy of first aid, the “head tilt” and “number of back blows” were more appropriate in the audiovisual group than in the auditory group. The audiovisual group had higher IP scores than the auditory group [8.23 ± 1.48 vs. 6.63 ± 2.36, *p* = 0.039]. Regarding the time required for first aid, no significant differences were observed in any items between the two groups (Table 5).

#### 3.4.2. Comparative Analysis of the Auditory and Audiovisual Groups Among Female Participants

Regarding the adequacy of first aid, the “infant position”, “supporting arm posture”, “head tilt”, and “hand part for back blows” were more adequate in the audiovisual group than in the auditory group. The audiovisual group had higher IP scores than the auditory group [8.12 ± 1.24 vs. 5.65 ± 2.13, *p* = 0.009]. Regarding the time required for first aid, no significant differences were observed in any items between the two groups (Table 5).

## 4. Discussion

FBAO is a leading cause of infant mortality, requiring prompt and appropriate first aid within 4 to 6 min to prevent serious consequences and potential mortality [11]. Effective interventions for FBAO are crucial in this time-sensitive emergency. Visual aids, such as images and illustrations, can help convey complex medical information and enhance communication [12]. And the adoption of video formats significantly augments comprehension compared to more traditional methods [7]. Our previous study demonstrated that using simple visual images, along with auditory guidance during dispatcher-assisted CPR, enhances bystander CPR performance [13]. This study investigated the application of these visual aids in infant FBAO first aid and found a significant improvement in performance (*p* < 0.001).

The treatment for infant FBAO entails the rescuer placing their forearm on their thigh, positioning the infant face-down on the forearm with the head lower than the chest, ensuring a straight neck, holding the chin, and administering five back blows between the shoulder blades. Then, the rescuer turns the baby’s face upward and applies pressure to the lower half of the sternum with two fingers five times, repeating these steps until the foreign body is expelled. In the event that the infant loses consciousness, infant CPR should be initiated as recommended [2,5].

The study revealed that the audiovisual group outperformed the auditory group in assessing the appropriateness of 10 infant FBAO first-aid items, particularly infant positioning, rescuer arm support posture, infant head tilting, and hand position for back blows. In the audiovisual guidance, participants successfully positioned the infant manikin to prevent it from slipping off their forearm. However, the proportion of correct hand positioning (chin support) was the lowest among participants in the audiovisual group, with no significant difference compared to the auditory group. This may be due to the difficulty of interpreting proper chin support from a small smartphone image.

Positioning the infant’s head lower than the body is emphasized in airway obstruction treatment guidelines because gravity aids in removing foreign objects. Research indicates that prone or head-down postures can help clear foreign bodies from the airway in both children and adults due to the effect of gravity [14,15]. In the audiovisual group, all participants correctly performed the infant head tilt maneuver, while only 30% of participants in the auditory-only group did so. Interestingly, the chest compression frequency was significantly lower in the audiovisual group compared to the auditory-only group (*p* = 0.03) because the participants in the audiovisual group performed more chest compressions than recommended. The process involved the transmission of two animated GIF images: one for back blows and another for chest compressions. After completing one cycle of audiovisual guidance, the participants were instructed to repeat the cycle until the foreign body was expelled. However, because the chest compression GIF remained visible on their smartphones, some participants continued to perform chest compressions instead of switching to back blows. This behavior was more pronounced in the group with no prior training. While a meta-analysis reported that videos could enhance CPR quality, concerns were raised regarding delays in initiating the first chest compression [16]. However, in this study, the use of animated GIFs for airway obstruction emergency treatment did not cause a significant delay in starting the first back blow between the audiovisual and auditory-only groups. The total execution time for three cycles was not significantly different between the two groups, suggesting that the images used in this study, consisting of only two images and transmitted quickly due to their small file size, allowed the users to understand the procedures intuitively without causing delays.

According to the most recent systematic review on this topic, while first aid is crucial for removing foreign objects, it can also pose a risk of injuries such as gastric rupture, splenic laceration, and pulmonary contusion in adults and children [17]. Infant first aid for FBAO is a complex procedure that involves constant manipulation of the infant’s position, making it challenging for dispatchers to convey verbally and for laypeople to understand and perform correctly [3,4]. Communication errors may contribute to these complications. Despite the simulation study being conducted in a controlled and quiet setting, video analysis revealed discrepancies in the auditory-only group. Three participants incorrectly used two fingers to press on the nipples instead of the center of the chest during chest compressions. Additionally, one participant applied pressure on the back with two fingers, while another used their elbow, all of which deviated from the prescribed instructions and constituted improper first aid practices. A Danish study that combined audio recordings of dispatcher-assisted CPR with closed-circuit television footage during out-of-hospital cardiac arrest scenarios revealed that verbal instructions given to bystanders are often misunderstood and can be unclear [18]. The challenge is compounded by physical, emotional, and knowledge barriers faced by lay responders in real-world emergencies [19,20]. A multicenter observational study in Japan found that proper first aid for airway obstruction was performed by bystanders only half of the time [21]. A literature review study has shown that biological and psychological stress experienced during CPR can lead to decreased performance ability [22]. Similar dynamics can apply in situations of infant airway obstruction, where parents or guardians, who are often the first responders, may experience significant mental and physical stress, hindering their ability to perform emergency procedures effectively. Numerous studies have reported that parents or guardians often lack the necessary emergency first-aid knowledge to handle crisis situations [23,24]. This study did not consider variables such as the participants’ occupations, education levels, or cultural backgrounds; however, in the comparison of sex differences, the audiovisual group significantly outperformed the auditory group in terms of IP scores. The female group demonstrated a higher number of significant performance scores than the male group (4 of 10 items vs. 2 of 10). Considering that in cases of infant FBAO, the primary witnesses are often parents, particularly young mothers, audiovisual aids could prove invaluable in facilitating effective first aid. Our findings suggest that individuals with no prior training achieved significantly higher compliance scores in the audiovisual group compared to other groups, indicating that simple animated images (GIFs) can effectively convey emergency first-aid knowledge to the general public.

With the global rise in smartphone penetration and advances in communication technology, there has been a surge in research utilizing video technology for CPR emergency interventions [8,25] However, research on applying such technology for airway obstruction first aid has been notably limited. Recent simulation studies in Japan that explored the use of video calls for infant airway obstruction first aid reported a 24% failure rate in video calls, highlighting various challenges in effectively employing real-time video during emergencies [3]. The transition from audio to video assistance requires the cooperation of the reporter, and issues related to the communication environment, communication costs, and exposure of personal information can pose significant barriers [9,26,27]. To address these challenges, researchers have developed text-transmitted animated GIF images with small file sizes, aiming to minimize delays in transmission and enhance usability. Therefore, using these GIFs during the EMS phase could offer cost-effective guidance for airway obstruction emergencies, regardless of the diverse communication environments worldwide.

As the proverb goes, “A picture paints a thousand words”, and visual communication is often more readily understood, particularly in emergencies. The authors developed small-packet animated GIFs to address communication barriers in the EMS phase, aiming to enhance the quality of emergency care for time-sensitive conditions and offer practical assistance in real-life situations. The issues highlighted in this study, such as visual readability and screen transitions, will be addressed systematically. Future research should focus on incorporating animated GIF images into real infant FBAO first-aid scenarios to assess their effectiveness.

This study has several limitations. First, due to the nature of simulation experiments, replicating real-world conditions outside of a hospital setting is challenging. Second, as this was a manikin-based simulation study, the actual effectiveness of using animated GIF images on real airway obstruction patients could not be assessed. Therefore, evaluating real-life outcomes, such as long-term follow-up, survival rates to discharge, or neurological outcomes, to assess the quality of the intervention was not possible. Third, the study participants were predominantly young and familiar with smartphones, which may limit the generalizability of the results to older age groups less accustomed to smartphones. Lastly, although it was a randomized controlled trial, blinding the evaluators was not feasible due to the nature of the study.

## 5. Conclusions

Simple animated GIFs have been developed and implemented to improve the quality of infant FBAO first aid in this study. Furthermore, this study demonstrated that combining simple animated images with auditory instructions can improve first-aid performance in groups without prior training experience.

## Figures and Tables

**Table 1:** Definitions of first-aid performance for infant choking.

	Assessment items	Definitions
Adequacy of	① Infant position	Prone for back blow and supine for chest thrust
	② Supporting arm posture	Alignment of the supporting arm and infant body axis
	③ Hand posture of the supporting arm	Adequacy of infant chin support
	④ Head tilt	The head is located lower than the body
	⑤ Location of back blows	Upper back above the axillary line
	⑥ Number of back blows	Back blow five times
	⑦ Hand part for back blows	Use the heel of hand
	⑧ Location of chest thrusts	The lower half of the sternum
	⑨ Number of chest thrusts	Chest thrust five times
	⑩ Repeating two maneuvers	Repeat the back blow and chest thrust maneuvers
Instruction Performance score		Sum of ①–⑩
Time to first back blow		Time from dispatcher instruction to the first back blow
Time to finish the first cycle (five back blows and five chest thrusts)		Time from dispatcher instruction to the fifth chest thrust
Time from the second to the third cycle		Time from the first back blow of the second cycle to when the participant finished the fifth chest thrust of the third cycle
Time to finish the third cycle		Time from dispatcher instruction to the fifth chest thrust of the third cycle

**Table 2:** General characteristics of the study participants compared.

		Auditory Group (*n* = 40)	Audiovisual Group (*n* = 39)	Total (*n* = 79)	*p*-Value
Sex	Male	19 (47.5%)	13 (32.5%)	32 (40.5%)	0.171
	Female	21 (52.5%)	26 (66.7%)	47 (59.5%)	
Age		30.43 ± 3.11	30.50 ± 2.47	30.46 ± 2.79	0.787
Prior training	No	25 (62.5%)	19 (48.7%)	44 (55.7%)	0.178
	Yes	15 (37.5%)	21 (53.8%)	36 (45.6%)	
Prior training timing (year)		4.88 ± 2.30	5.67 ± 2.46	5.32 ± 2.39	0.310

**Table 3:** Comparative analysis of auditory and audiovisual groups of first-aid performance for infant choking.

	Assessment Items	Auditory (*n* = 40)	Audiovisual (*n* = 39)	*p*-Value
Adequacy of	① Infant position	31 (77.5%)	39 (100.0%)	**0.002**
	② Supporting arm posture	12 (30.0%)	27 (69.2%)	**<0.001**
	③ Hand posture of the supporting arm	10 (25.0%)	15 (38.5%)	0.233
	④ Head tilt	12 (30.0%)	39 (100.0%)	**<0.001**
	⑤ Location of back blows	22 (55.0%)	31 (79.5%)	0.138
	⑥ Number of back blows	29 (72.5%)	34 (87.2%)	0.105
	⑦ Hand part for back blows	19 (47.5%)	31 (79.5%)	**0.009**
	⑧ Location of chest thrusts	32 (80.0%)	30 (76.9%)	0.136
	⑨ Number of chest thrusts	40 (100.0%)	34 (87.2%)	**0.026**
	⑩ Repeating two maneuvers	36 (90.0%)	38 (97.4%)	0.175
Instruction Performance score		6.13 ± 2.27	8.15 ± 1.31	**<0.001**
Time to first back blow		52.88 ± 12.56	53.05 ± 10.95	0.498
Time to finish the first cycle		77.38 ± 14.03	112.58 ± 144.38	0.118
Time from the second to the third cycle *		19.31 ± 4.40	22.29 ± 4.64	0.332
Time to finish the third cycle *		112.53 ± 17.99	128.76 ± 17.64	0.332

* The analysis was conducted on the auditory (n = 36) and audiovisual (n = 38) groups, excluding cases in which the two maneuvers were not performed repeatedly.

**Table 4:** Comparative analysis of auditory and audiovisual groups of first-aid performance for infant choking by prior training experience.

		Prior Training Group			No Prior Training Group		
		Auditory (*n* = 15)	Audiovisual (*n* = 20)	*p*-Value	Auditory (*n* = 25)	Audiovisual (*n* = 19)	*p*-Value
Adequacy of	① Infant position	13 (86.7%)	20 (100.0%)	0.093	18 (72.0%)	19 (100.0%)	**0.012**
	② Supporting arm posture	8 (53.3%)	12 (60.0%)	0.693	4 (16.0%)	15 (78.9%)	**<0.001**
	③ Hand posture of the supporting arm	6 (40.0%)	7 (35.0%)	0.668	4 (16.0%)	8 (42.1%)	0.054
	④ Head tilt	5 (33.3%)	20 (100.0%)	**<0.001**	7 (28.0%)	19 (100.0%)	**<0.001**
	⑤ Location of back blows	11 (73.3%)	16 (80.0%)	0.621	11 (44.0%)	15 (78.9%)	0.079
	⑥ Number of back blows	9 (60.0%)	18 (90.0%)	**0.036**	20 (80.0%)	16 (84.2%)	0.720
	⑦ Hand part for back blows	10 (66.7%)	18 (90.0%)	0.088	9 (36.0%)	13 (68.4%)	0.059
	⑧ Location of chest thrusts	14 (93.3%)	17 (85.0%)	0.129	18 (72.0%)	13 (68.4%)	0.298
	⑨ Number of chest thrusts	15 (100.0%)	18 (90.0%)	0.207	25 (100.0%)	16 (84.2%)	**0.040**
	⑩ Repeating two maneuvers	13 (86.7%)	19 (95.0%)	0.383	23 (92.0%)	19 (100.0%)	0.207
Instruction Performance score		6.93 ± 2.15	8.25 ± 1.33	0.263	5.63 ± 2.24	8.05 ± 1.31	**0.014**
Time to the first back blow		50.13 ± 9.50	52.85 ± 12.10	0.559	54.52 ± 14.01	53.26 ± 9.95	0.256
Time to finish the first cycle		74.73 ± 11.48	132.29 ± 199.14	0.128	78.96 ± 15.36	9.079 ± 12.32	0.670
Time from the second to the third cycle *		19.38 ± 3.89	22.53 ± 4.95	0.490	19.26 ± 4.75	22.05 ± 4.43	0.492
Time to finish the third cycle *		111.00 ± 17.45	128.58 ± 20.86	0.285	113.96 ± 18.52	128.95 ± 14.30	0.755

* The analysis was conducted on only those participants who properly performed “Repeating two maneuvers (⑩)”.

**Table 5:** Comparative analysis of auditory and audiovisual groups of first-aid performance for infant choking by sex.

		Male			Female		
		Auditory (*n* = 19)	Audiovisual (*n* = 13)	*p*-Value	Auditory (*n* = 21)	Audiovisual (*n* = 26)	*p*-Value
Adequacy of	① Infant position	15 (78.9%)	13 (100.0%)	0.081	16 (76.2%)	26 (100.0%)	**0.008**
	② Supporting arm posture	8 (42.1%)	7 (53.8%)	0.529	4 (19.0%)	20 (76.9%)	**<0.001**
	③ Hand posture of the supporting arm	6 (31.6%)	6 (46.2%)	0.419	4 (19.0%)	9 (34.6%)	0.294
	④ Head tilt	10 (52.6%)	13 (100.0%)	**0.002**	2 (9.5%)	26 (100.0%)	**0.001**
	⑤ Location of back blows	11 (61.1%)	11 (84.6%)	0.165	11 (61.1%)	20 (74.1%)	0.374
	⑥ Number of back blows	14 (73.7%)	12 (92.3%)	**0** **.005**	15 (71.4%)	22 (84.6%)	0.282
	⑦ Hand part for back blows	11 (61.1%)	11 (84.6%)	0.165	8 (40.0%)	20 (76.9%)	**0.** **029**
	⑧ Location of chest thrusts	15 (93.8%)	11 (91.7%)	0.840	17 (89.5%)	19 (73.1%)	0.264
	⑨ Number of chest thrusts	19 (100.0%)	11 (84.6%)	0.082	21 (100.0%)	23 (88.5%)	0.112
	⑩ Repeating two maneuvers	17 (89.5%)	12 (92.3%)	0.840	19 (90.5%)	26 (100.0%)	0.112
Instruction Performance score		6.63 ± 2.36	8.23 ± 1.48	**0.039**	5.65 ± 2.13	8.12 ± 1.24	**0.009**
Time to the first back blow		53.68 ± 15.11	53.31 ± 9.54	0.294	52.14 ± 10.06	52.92 ± 11.77	0.830
Time to finish the first cycle		78.79 ± 17.10	93.38 ± 10.68	0.304	76.10 ± 10.82	121.81 ± 17.91	0.124
Time from the second to the third cycle *		19.58 ± 3.63	24.00 ± 4.64	0.163	18.05 ± 5.36	21.35 ± 4.39	0.926
Time to finish the third cycle *		115.24 ± 21.60	134.75 ± 15.91	0.311	110.11 ± 14.19	126.00 ± 18.0	0.301

* The analysis was conducted on only those participants who properly performed “Repeating two maneuvers (⑩)”.

## Data Availability

The datasets analyzed in this study are available from the corresponding author upon reasonable request.

## Abbreviations

The following abbreviations are used in this manuscript:

FBAO	Foreign body airway obstruction
IP	Instruction Performance
GIFs	Graphics interchange formats

## Appendix A

**Table A1:** Protocol for auditory and audiovisual groups in first aid of airway obstruction for infants.

Auditory group
▪ Airway obstruction is suspected, and first aid is required. ▪ Change your smartphone to speakerphone mode and place it somewhere visible. ▪ Remove clothing to expose your infant’s chest. ▪ Turn the infant over and place their stomach and chest on one of your arms. Firmly grasp the infant’s chin and neck with the palm of the same arm. ▪ In this position, lift the infant so that their face is facing the floor, tilt your arm so that the head is slightly downward, and firmly tap the infant’s back between the shoulder blades with the heel of your other hand five times in a row. ▪ Turn your infant so that their face is facing upward. ▪ Using your second and third fingers, quickly press five times to a depth of 2.5 cm on the center of the chest between both nipples. ▪ Alternate between these two treatments until the foreign object is dislodged. ▪ If your infant loses consciousness during the process, please notify us immediately.
Audiovisual group
▪ Airway obstruction is suspected, and first aid is required. ▪ Change your smartphone to speakerphone mode and place it somewhere visible. ▪ I just sent you a text message, so please click on the link right away. Do you see the picture? ▪ Just follow the picture. ▪ Remove clothing to expose your infant’s chest. ▪ As shown in the picture, turn the infant over and place the infant’s stomach and chest on one of your arms, and firmly grasp the infant’s chin and neck with the palm of the same arm. ▪ In this position, lift the infant so that their face is facing the floor, tilt your arm so that the head is slightly downward, and firmly tap the infant’s back between the shoulder blades with the heel of your other hand five times in a row. ▪ Then, turn your infant so that their face is facing upward. ▪ I just sent you another text, please check. You see the picture, right? ▪ As shown in the picture, using your second and third fingers, quickly press five times to a depth of 2.5 cm on the center chest area between both nipples. ▪ Continue alternating between these two treatments until the foreign object is dislodged. ▪ If your infant loses consciousness during this process, please notify us immediately.
